# Epidemiology of eating disorders, eating disordered behaviour, and body image disturbance in males: a narrative review

**DOI:** 10.1186/s40337-015-0058-y

**Published:** 2015-05-23

**Authors:** Deborah Mitchison, Jonathan Mond

**Affiliations:** School of Medicine, University of Western Sydney, Sydney, Australia; Department of Psychology, Faculty of Human Sciences, Macquarie University, Sydney, Australia; Research School of Psychology, Australian National University, Sydney, Australia

**Keywords:** Epidemiology, Sex, Gender, Eating disorder, Muscle dysmorphia, Quality of life, Help-seeking, Review, Prevalence

## Abstract

Challenges to epidemiological studies of eating and related body image disturbance disorders in males include, in addition to low base rates and the predominance of residual diagnostic categories, the female-centric nature of current classification schemes and the consequent lack of appropriate assessment instruments. In this narrative review, we summarise epidemiological data regarding the prevalence and correlates of eating disorders, related body image disturbance disorders, and eating disorder features in males. Attention is focused on disorders most likely to be observed among males, such as muscle dysmorphia and muscularity-oriented excessive exercise. It is argued that, given the multiple challenges involved in research of this kind, a focus on features is more likely to advance the field than a focus on diagnoses. In terms of correlates, we focus on impairment and help-seeking, since these issues are most relevant in informing public health burden, service provision, and related issues. We end with some thoughts about current gaps in the knowledge base and directions for future research that we consider to be most promising.

## Challenges to epidemiological studies of eating disorders in males

A number of challenges plague the study of eating disorder epidemiology, limiting particularly our understanding of the prevalence and correlates of these disorders in males. Not least has been the dominance of the DSM-IV (Diagnostic and Statistical Manual of Mental Disorders [1]) residual diagnostic category, Eating Disorder Not Otherwise Specified (EDNOS). Aside from the irony of a residual category constituting the majority of cases, the heterogeneity of EDNOS has meant that prevalence studies have often yielded little descriptive information regarding the behavioural and cognitive profile of eating disorders in the population. This may be a relatively greater problem for males, with a general population study in the United States for instance having found that 83 % of male versus 71 % of female eating disorder cases could be classified as EDNOS [2]. Recent changes to this classification in DSM-5 [3] will likely ameliorate, but not resolve, this problem (e.g., [4-7]).

A related issue is the low population prevalence of eating disorders meeting formal diagnostic criteria, even in females, which necessitates large samples in order to detect cases. However, eating disorders have often been excluded from national mental health surveys (e.g., [8-10]), or their inclusion is limited to anorexia nervosa (AN) and/or bulimia nervosa (BN; e.g., [11-21]). Given that ‘other’ eating disorders, including binge eating disorder (BED), comprise the majority of community male cases [22], their exclusion from large-scale surveys is problematic when drawing conclusions as to prevalence.

The problem of low base rates is compounded by the problem of non-response bias, namely, the known tendency for individuals with eating and other mental disorders to be over-represented among non-respondents in epidemiological studies [23,24]. The factors that account for this bias, such as lack of insight, denial, shame and secrecy, apply to studies of eating disorders in both women and men. However, their influence may be greater in studies of males because insight may be particularly poor, and perceived stigma associated with disclosure of mental health problems may be particularly salient [25,26].

Other methodological problems that have limited the interpretation and comparability of findings across previous epidemiological studies include the variable selection of samples in terms of age range, ethnicity, and geographic location; the variable classification schemes applied over time (e.g., DSM-III, DSM-IV, DSM-5); and the variable methods of assessment adopted (e.g., structured diagnostic interview vs. self-report questionnaire). These problems are inherent, more or less, in epidemiological studies of all mental health problems but again may be particularly problematic in population-based research addressing the prevalence and correlates of eating-disordered behaviour in males.

Finally, and perhaps most importantly, there remains the issue of eating disorders being conceptualised, historically, as a problem of young females and, in turn, classification schemes and assessment methods being developed with this premise in mind [22]. The inclusion of BED as a formal diagnosis and removal of the amenorrhea criteria in AN in the DSM-5 are a step forward in this regard, but it is apparent that making the DSM less “female-centric” was not a priority of the DSM-5 Eating Disorders Working Group [7]. Thus, although we are currently in a climate of increased appreciation of eating and body image problems in males, our methods of identification, assessment, classification, and treatment are yet to catch up.

### Scope of the current contribution

The goal of the current contribution is to provide a narrative summary of the literature to date bearing on the prevalence and correlates of eating and related body-image disorders, and of eating-disordered behaviour, in males. The goal was not to conduct a systematic review of epidemiological studies of eating disorders in males. Our view is that, given the challenges outlined above, recent changes to the DSM classification of eating disorders, and the relative infancy of epidemiological studies of eating disorders in males, such an endeavour would be of limited benefit. With respect to correlates, we focus on functional impairment and help-seeking behaviour since considerations in this regard have particular relevance to issues of public health burden and service provision. Further, these issues have often been neglected in previous reviews (e.g., [27,28]). We end with some thoughts about priorities for future research in this field.

### Eating disorder prevalence

#### Adults

A review of DSM-5 eating disorder prevalence studies in males was recently conducted by Raevuori and colleagues [29]. In regards to the major diagnostic entities, BED is the most prevalent in adult males, with estimates of lifetime prevalence of 0.78 % [30], 1.55 % [31,32], and 2.0 % [2,33]. Estimates of the lifetime prevalence of BN and AN are lower and more variable, between 0.13 % and 1.34 % for BN [2,11-13,30-35], and 0.00 % and 0.53 % for AN [2,11-13,18,30-33,35,36]. As noted above, the DSM-IV residual category of EDNOS has accounted for the majority of male eating disorders, with one study reporting a lifetime prevalence of 3.38 % in adult males [2]. Population prevalence estimates for the DSM-5 residual categories of Other Specified Feeding and Eating Eisorders (OSFED) and Feeding and Eating Disorders Not Elsewhere Classified (FEDNEC) are not yet available. The lifetime prevalence rates for all eating disorders have repeatedly been found to be lower in males (for a review of demographic correlates see [37]), although studies in the United States and the Netherlands have also found evidence of no sex differences [11,12,30]. The latter studies however identified few eating disorder cases, and thus had low statistical power to detect such differences. The hierarchical pattern of diagnosis mirrors that seen amongst females, with EDNOS and BED being more prevalent than BN, which in turn is more prevalent than AN.

#### Adolescents

Researchers have recently begun to conduct rigorous studies of the epidemiology of eating disorders in adolescents. The majority of these studies have found the lifetime or current prevalence of disorders meeting formal diagnostic criteria for AN (0 – 0.2 %), BN (0 – 0.7 %), and BED (0 – 0.8 %) to be low in adolescent males [4,15, 21,38-45]. A recent study that amalgamated full and partial syndrome eating disorders reported point prevalence rates among 11 to 18 year-old boys of 0.2 % for full or partial BN, and 0.4 % for full or partial BED [46]. The higher proportion of identified eating disorder cases in adult versus adolescent males may be indicative of a later onset of eating disorders in males, as compared to females where prevalence tends to peak before age 25 [27,33]. This is supported by a recent study, which found that males hospitalised with AN had a later age of onset than females [47]. However, other population-based data suggests that there are no sex differences in age of onset [34], including for early (<14 years) onset cases [48,49].

### Eating disorder symptoms

Investigating eating disorder epidemiology at the symptom or feature level may be particularly important in males [50]. In addition to the issues discussed above, most eating disorder features are transdiagnostic across the eating disorders (e.g., binge eating may present in AN, BN, and BED), which results in frequent diagnostic migration and limited utility of diagnostic-level epidemiological data [51]. Further, we are still a long way from understanding how behavioural, emotional, and cognitive sequelae may cluster together to constitute eating disorders in men. However, we do know that eating disorder behaviours and associated cognitive features, such as the overvaluation of weight or shape, are associated with high levels of distress and disability in males (and females) regardless of whether they occur in isolation or in conjunction with the other behaviours or features required for specific diagnoses. Indeed, the distinction between “eating disorders” and “eating-disordered behaviour” has become increasingly blurred with the inclusion of BED as a formal diagnosis and with the suggestion that other syndromes characterised primarily by a single eating disorder behaviour, such as “purging disorder”, may also be “clinically significant”. Thus, a focus on the prevalence and correlates of eating disorder features should play an important role in future epidemiological studies of both males and females.

Recent epidemiological studies that have provided prevalence estimates of specific eating disorder features and symptoms, findings from which are outlined below, include cross-sectional surveys of the adult population in Germany (N = 2520; age ≥ 14 years) [52] and the United States (“National Comorbidity Survey Replication”; N = 2980; age ≥ 18 years) [33] and sequential cross-sectional surveys of the South Australian adult population (“Health Omnibus Survey”; N = 3001 – 3047; age ≥ 15 years) [53-55]. Other studies cited include cross-sectional surveys of 20 – 44 year-olds in the general Australian population [56], United States employees (age 18 – 65 years) [57], 18 – 35 year-olds in the United States on a Health Plan [58], and Australian Capital Territory (ACT) high school students [59].

#### Binge eating

Binge eating appears to be the most common eating disorder behaviour in males, with a close to equal prevalence to females. The prevalence of any objective binge eating over the past month was 4.2 % for both males and females in the German study [52] and 7.5 % for males (vs. 11.2 % of females) in the United States employee survey [57]. Estimates of the prevalence of at least weekly episodes of objective binge eating (the threshold for DSM-5 diagnosis of BED and BN) have also varied considerably, from 0.9 % of males in the German study [52] to 7.8 % [54], and 4.1 % [55], respectively, in the 2005 and 2008 Health Omnibus Surveys, and 5.1 % in the Australian survey of 20 – 44 year-olds [56] (these figures were 1.4 %, 7.5%, 5.7 %, and 9.9 %, respectively, among female participants). In the National Comorbidity Replication Survey, the prevalence of twice-weekly objective binge eating (the threshold for DSM-IV diagnosis) for a period of 3 months in the past year was 1.7 % in males, which was not significantly lower than for females (2.5 %) [33]. However, when excluding individuals who met criteria for formal eating disorders (termed sub-threshold binge eating disorder by the authors), males were three times as likely as females to report this frequency of binge eating. In regards to adolescents, the ACT high school survey found that 6.0 % of male students (and 16.6 % of females) engaged in at least weekly objective binge eating over the past month [59]. In terms of time trends, comparisons of the Health Omnibus Surveys found that the prevalence of objective binge eating increased significantly in males from 1995 to 2005 [54] and from 1998 to 2008 [50].

Less is known about the prevalence of subjective binge eating episodes in males, despite strong evidence, in adolescent and young adult females at least, that the experience of loss of control over eating is a better predictor of eating disorder and comorbid psychopathology than the amount of food consumed [60]. The occurrence of objective binge eating episodes remains a requirement for the diagnoses of BN and BED in the DSM-5, whereas subjective binge eating may be sufficient for these diagnoses in the upcoming revision of the International Classification of Diseases (ICD-11 [61]). The studies to date that have assessed loss of control over eating outside of objective binge eating suggest that this behaviour is more common in women and girls. For instance, in the survey of 18–35 year-olds on a Health Plan in the US, more women than men reported “often” feeling a sense of loss of control whilst eating [58]. Further, approximately 1 % of men versus 2.8 % of women in the 2008 Health Omnibus Survey [55] and 3.4 % of boys versus 12.3 % of girls in the ACT high school survey [59] reported at least weekly episodes of subjective binge eating.

#### Extreme dietary restriction and purging

While the use of extreme weight-control behaviours to achieve weight loss has historically been assumed, and found, to be largely confined to women, findings from recent studies suggest that sex differences in the prevalence of these behaviours may be diminishing [50]. In the German study, 7.7 % of men (11.9 % of women) had engaged in extreme caloric restriction at least once over the past month [52]. Weekly occurrence of extreme dietary restriction on the other hand was reported by 3.9 % and 2.4 % of men (5.2 % and 4.3 % of women), respectively, in the 2005 [54] and 2008 [55] Health Omnibus Surveys, and 1.4 % of men (2.4 % of women) in the German study [52]. Interestingly, fasting at least three times per week over the past month was reported by 1.9 % of males in the Australian survey of 20 – 44 year-olds, which was not significantly different from the proportion of females (2.4 %) reporting the same behaviour [56]. Similar rates of at least thrice-weekly episodes of extreme dietary restriction were reported in adolescent males in the ACT high school survey (2.3%), however the rate was considerably higher among female students (11.5 %) in this study.

In the German study, 0.4 % of men (1.3 % women) reported self-induced vomiting and 0.8 % of men (2.4 % of women) reported using laxatives at least once over the past month [52]. These rates reduced to 0.1 % (women: 0.3 %) for self-induced vomiting and 0.2 % (women: 0.7 %) for laxative use when considering occurrence on at least four occasions over the past month. On the other hand, 1.5 % of 20 – 44 year-old males (4.0 % of females) in the Australian population survey reported having purged at least once in the past month [56]. In the Health Omnibus Surveys, at least weekly episodes of purging (self-induced vomiting, laxative, or diuretic use) were reported by 1.0 % of men in 2005 [54] and 0.5 % in 2008 [55] (women: 2.1 % and 1.5 %, respectively). At least weekly self-induced vomiting was reported by 0.8 % of males (vs. 3.3 % of females) in the ACT high school survey [59].

Comparing data from the 1998 and 2008 Health Omnibus Surveys, while the prevalence of extreme dietary restriction and purging remained more common in females, these behaviours increased at a faster rate in males during the 10-year period [50]. When compared with 1998 figures, males in 2008 were five times more likely to engage in both extreme dietary restriction (females less than twice as likely) and purging (females slightly less likely) on a regular basis.

#### Overvaluation of body weight and/or shape

In terms of cognitive features, the Australian population survey of 20 – 44 year-olds found that 3.9 % of men in this age group reported overvaluation of weight/shape [56] and in the 2008 Health Omnibus Survey 13.5 % of males reported overvaluation [55]. While these figures may appear surprisingly high, overvaluation of weight and/or shape was significantly higher amongst females in both of these studies (14.4 % and 23 %, respectively). On the other hand only 4.9 % of boys reported overvaluation of body weight or shape in the ACT high school study, compared to 24.2 % of girls [59], supporting the notion that disturbances in eating and body image may have a later onset in males.

### Muscle dysmorphia

A disorder that is gaining increasing attention from researchers interested in the epidemiology of eating-disordered behaviour in males is muscle dysmorphia. Although this condition is currently classified as a subtype of body dysmorphic disorder, within the Obsessive Compulsive and Related Disorders section of DSM-5, some authorities have argued that it would be more appropriately classified as an eating disorder [62,63]. Specifically, it has been suggested that muscle dysmorphia may be the male equivalent to AN [64], exhibiting a relatively higher prevalence in males as opposed to females, and characterised by a drive for muscularity as opposed to thinness. In support of this view, muscle dysmorphia has consistently been found to be associated with eating disorder symptoms [65-67], as well as neurocognitive deficits similar to those seen among individuals with AN [68]. Further, muscle dysmorphia appears to be distinct from other forms of body dysmorphic disorder, in that male patients with muscle dysmorphia have greater levels of psychopathology, psycho-social impairment, and suicide risk, compared to male patients with other forms of body dysmorphic disorder [69]. Incorporation of muscle dysmorphia and, perhaps, variants of this disorder, in classification schemes for eating disorders would be one way to address the issue of these schemes being unduly female-centric [7].

Currently, little is known about the population prevalence of muscle dysmorphia, due in part to a lack of consensus regarding appropriate diagnostic criteria. In those few studies that have investigated the population prevalence of body dysmorphic disorder, exclusions have been applied, such as concerns with overall shape and/or weight, in order to exclude eating disorders. This has inadvertently resulted in cases of muscle dysmorphia being missed (e.g., [70]). For instance, in a study of the German population, Rief and colleagues reported a prevalence rate for body dysmorphic disorder in adult males of 1.4 %. However, this excluded the 2.5 % of male participants who had an excessive primary appearance concern with their weight [71]. The literature specifically investigating muscle dysmorphia has been confined largely to male university and male gymnasium-attending samples. Problems with this include: non-university attending males with muscle dysmorphia are not represented; muscle dysmorphia likely affects a broader demographic than is typically represented in college samples; a proportion of people with muscle dysmorphia may avoid exercise in gymnasiums or other public facilities; and the fact that muscle dysmorphia may also present, albeit less commonly, in women.

#### Excessive/compulsive exercise

Arguably the behaviour most relevant for males with body image and/or eating disturbances, including those with muscle dysmorphia, is compulsive or excessive exercising (including excessive efforts to increase muscle bulk and/or definition). The German and Australian (20 – 44 year-olds) population surveys reported that 3.5 % [52] and 3.2 % [56] of adult males engaged in driven or excessive exercise in the past month, respectively. Excessive exercise may be more prevalent in younger cohorts with 5.6 % of males in the survey of 18–35 year-olds on a Health Plan in the USA reporting “often” exercising to lose weight following a binge eating episode [58] and 5.3 % of males in the ACT high school sample reporting regular compulsive exercise over the past month [59]. In all studies, no sex differences in the proportion of participants who reported these behaviours were observed. Further, all of these surveys employed measures tapping weight-loss-related excessive exercise as prompted by a drive for thinness. The assessment of pathological exercise in males has been hampered by under-recognition of body image and eating disturbances that stem from a drive for muscularity rather than thinness [59]. Recent years have witnessed the development of measures of “drive for muscularity” and related constructs (e.g., [72]). The inclusion of these measures in future research should facilitate efforts to provide a more reliable assessment of the prevalence and correlates of excessive exercise in males [59].

#### Body dysmorphic behaviours

Other behaviours that may be relevant to males with eating and related body image problems, particularly muscle dysmorphia, include compulsive symptoms typically linked to body dysmorphic disorder, such as: excessive body checking (e.g., looking at one’s reflection in the mirror/window, fat pinching, weighing, etc.); body avoidance (e.g., deliberately covering or not looking in mirrors, avoiding knowledge of weight etc.); camouflaging (e.g., wearing baggy clothing to hide the contours of the body, positioning oneself to show less body); reassurance-seeking, making comparisons to others’ bodies; and social avoidance [73]. Epidemiological studies of these behaviours have not yet been conducted, in males or females, despite preliminary evidence that they are strongly associated with eating disorders in women [74] and may be highly prevalent in males. For instance, in the survey of 18–35 year-olds on a Health Plan in the USA, 8.9 % of males reported frequent body checking and 4.4 % of males reported frequent body avoidance [58].

#### Other muscle dysmorphic behaviours

Concerning other behaviours likely to be associated with muscle dysmorphia, there is no population-based epidemiological data to report. In a twin study conducted in Finland, 12 % of males reported the use of supplements and/or steroids in order to influence their body build, and this proportion was considerably higher amongst males who reported marked muscular dissatisfaction [75]. In other studies using university- and gymnasium-attending samples, muscle dysmorphia has been found to be associated with increased body-building, steroid use [65-67], and strict adherence to a high protein diet [76].

#### Muscular dissatisfaction

Rather than being preoccupied with thinness, males are more likely to be preoccupied with body composition (i.e. fat to muscle ratio). For instance, the Finnish twin study above found an inverted-U relationship between subjective wellbeing and body mass index in males, demonstrating that, unlike females, males were most satisfied with a higher (but not obese) body weight [77]. Dissatisfaction with muscularity appears to be extremely common among males, with up to 85 % of male university students in France reporting some level of dissatisfaction with their musculature [78] and 29.9 % of 22–27 year-old males in the Finnish twin study reporting that they were highly dissatisfied with their musculature [75].

### Impairment in role and/or psycho-social functioning

Epidemiological studies of eating disorders have rarely assessed impairment and in at least some of the studies that have done so only females have been included or relevant data have not been stratified by sex (e.g., [21,33]). Further, the challenges of low base rates of eating disorders meeting formal diagnostic criteria and non-response bias have proven particularly problematic when considering impairment, since the number of participants with current disorders has frequently been too small to permit meaningful analysis of these correlates, even in females. A large, population-based study in Canada found no differences in quality of life impairment among males and females with full or partial syndromes of AN or BN [34]. Comorbidity with other mental health problems was also similar between these men and women, with the exceptions that alcohol dependence was more common among males with eating disorders and major depression was more common among females with eating disorders.

To our knowledge, the only adult population-based research to have examined sex differences in impairment associated with eating-disordered behaviour was that conducted by the authors and colleagues using data from the Australian population survey of 20 – 44 year-olds [56] and the South Australian Health Omnibus Surveys conducted in 2005 and 2008 [55,79]. In the survey of 20 – 44 year-olds, extreme dietary restriction, objective binge eating, purging, and overvaluation of body weight and/or shape were all associated with significantly lower life satisfaction and social support and significantly greater psychological distress and this was the case in both males and females [56]. Further, the level of impairment associated with these features did not differ significantly between males and females, with the exception that extreme dietary restriction was associated with greater distress in women. In the 2005 Health Omnibus Survey, men who experienced regular objective binge eating or extreme dietary restriction reported a significantly higher number of days out of role (DOR; e.g., inability to attend to work, school, or other duties) in the past month than men who did not report these behaviours (binge eating: 2.6 vs. 1.8, extreme dietary restriction: 4.0 vs. 1.8). This number was higher still for those men who also endorsed overvaluation of body weight/shape [79]. No such relationship was observed for men who reported episodes of purging, although statistical power for this comparison was constrained by the small number of males reporting this behaviour (n = 14, 1.1 %). The only sex differences in the associations between eating disorder behaviours and impairment observed in this study were that both behavioural and cognitive eating disorder features predicted DOR in men whereas only the cognitive feature of overvaluation of body weight/shape predicted DOR in women. There was also little evidence for sex differences in quality of life impairment associated with eating disorder behaviours in the 2008 Health Omnibus Survey, the only exceptions being that objective binge eating was associated with greater impairment in quality of life in men while the overvaluation of weight/shape was associated with greater impairment in quality of life in women [55].

In terms of impairment associated with eating disorder features reported by adolescents, the ACT high school study found that each of the eating disorder features assessed – objective binge eating, subjective binge eating, self-induced vomiting, laxative misuse, extreme dietary restriction, excessive exercise, and overvaluation of weight or shape – was associated with elevated levels of both general psychological distress and quality of life impairment in both male and female students [80]. Further, and consistent with findings from the adult studies, levels of impairment associated with these features did not differ significantly by sex, with the exception that subjective binge eating was associated with greater impairment in females.

Little is known about impairment in psycho-social functioning among individuals with muscle dysmorphia. In the Finnish twin study by Raevouri and colleagues [75], a linear relationship was found between muscular dissatisfaction and life satisfaction, such that males with marked muscle dissatisfaction had the lowest levels of life satisfaction. A linear relationship between weight/shape dissatisfaction and quality of life impairment was similarly observed in a large, general population study of women [81]. These findings suggest that, in terms of their impact on quality of life, the effects of muscularity-oriented body dissatisfaction in men may be comparable to fat-oriented body dissatisfaction observed in women.

### Help-seeking

Virtually nothing is known about the proportion of males with eating or related disorders who seek help for such problems, or for comorbid physical or mental health problems, and, if so, from whom [80]. Again, low base rates, non-response bias, and female-centric classification schemes and assessment measures have precluded meaningful investigation of help-seeking behaviour among males with these conditions.

In the only study of this kind we were able to identify, a random sample of male patients presenting to two general practices completed a screening questionnaire and those identified as having eating disorder symptoms were invited to complete a structured interview. Of 6 participants (1.6 %) who met criteria for a lifetime DSM-IV eating disorder diagnosis based on the interview assessment, 1 (16.7 %) had sought treatment for an eating disorder [82]. Unfortunately, help-seeking behaviour was not assessed in the larger group of 75 men (20 % of the total sample) who were identified as having eating disorder symptoms according to the screening questionnaire. Nonetheless, this study provides preliminary evidence to support the widely held view that uptake of mental health care among males with eating disorders is probably even lower than that of females with these disorders, the latter estimated to be, on average, approximately 25 % [83].

A study that assessed the number of male admissions to an eating disorder inpatient unit in the United States found that the number of admissions had increased significantly over the period from 1984 to 1997 [84]. The ratio of males to females, however, remained small and information concerning the proportion of males with eating disorders in the community who received inpatient treatment was not available.

A recent qualitative study of males with eating disorders who had sought help for an eating problem found that men tend to present to treatment services late in the trajectory of their illness [85], a theme that has emerged in other qualitative studies [86]. Although it is unclear whether delays in help-seeking among males with eating disorders exceed those observed in females, it is reasonable to posit that low uptake of mental health care and late presentation to treatment for males with eating disorders reflect, at least in part, increased stigma associated with what is considered by many to be a feminine problem [25,26,87]. However, this may not hold true for disorders characterised by more masculine ideals. For instance, a recent study that investigated attitudes toward people with body image disorders reported less stigmatisation toward a description of a male character with muscle dysmorphic disorder as compared to a male character with AN [26]. Another qualitative study that interviewed multiple stakeholders, including eating disorder organisations, clinical specialists, and men with eating disorders, concluded that health professionals should receive training in the detection of eating disorders in males and that service providers should adapt their services to be more ‘male-friendly’ [88].

### Review and Conclusion

In sum, while the current state of epidemiological research precludes an understanding of the full burden of eating and related disorders in males, there is good evidence that males, like females, suffer from disturbances in body image, binge eating, and maladaptive weight/shape control behaviours. Indeed, the prevalence of binge eating may be nearly as high in males as in females and the prevalence of extreme weight control behaviours, such as extreme dietary restriction and purging, may be increasing more rapidly in males than females. Although it is likely that the population prevalence of features such as muscularity-oriented excessive exercise, steroid abuse, and muscularity-oriented dissatisfaction and overvaluation is higher amongst males, this has not yet been systematically investigated. Finally, the impairment in quality of life, lost productivity, and psychosocial wellbeing associated with eating disorders and related disturbances appears to be comparable in males and females. It is thus vital that barriers to help-seeking, such as stigma, ignorance more generally, and female-centric services, are addressed.

### Future directions

It is apparent that future epidemiological studies need to be more inclusive of males and male-relevant variables. New data is required to inform the prevalence of DSM-5 eating disorders in both males and females. Arguably, research focusing on behaviours, rather than diagnostic entities, is best placed to advance our understanding of the epidemiology of eating and related disorders in males. An approach of this kind effectively controls for changes in diagnostic criteria over time and, moreover, has the potential to inform refinements in this regard. Existing measures will need to be modified to better assess, and screen for, muscularity-oriented disordered eating and body image disturbance and/or new measures will need to be developed for this purpose. Population- and/or primary-care-based research is urgently needed to inform health service utilisation and the factors associated with this among males with eating and other body image disturbance disorders. The factors considered will need to include traditional determinants of help-seeking for mental health problems, such as the cost of and availability of treatment, but also “mental health literacy variables” such as self-recognition of problem behaviour where this exists and perceived need for treatment [87]. Research of this kind will be increasingly feasible in light of changes to classification schemes, improved community eating disorders mental health literacy, and lower perceived stigma associated with disclosure of eating disorder symptoms and other mental health problems among both women and men. In the meantime, strategies designed to circumvent the problem of low base rates employed in early studies of the epidemiology of eating disorders in females, such as the use of two-phase designs and over-sampling of high-risk populations (e.g., [24]) will likely be needed.

## Abbreviations

ACT: Australian Capital Territory
AN: Anorexia nervosa
BED: Binge eating disorder
BN: Bulimia nervosa
DOR: Days out of role
DSM: Diagnostic and Statistical Manual of Mental Disorders
EDNOS: Eating disorder not otherwise specified
FEDNEC: Feeding and eating disorder not elsewhere classified
ICD: International Classification of Diseases
OSFED: Other specified feeding and eating disorder
